# Differences in the histological findings, phenotypic marker expressions and genetic alterations between adenocarcinoma of the gastric cardia and distal stomach

**DOI:** 10.1038/sj.bjc.6603583

**Published:** 2007-01-30

**Authors:** Y Tajima, K Yamazaki, R Makino, N Nishino, Y Masuda, S Aoki, M Kato, K Morohara, M Kusano

**Affiliations:** 1 Division of General and Gastroenterological Surgery, Department of Surgery, Showa University, School of Medicine, 1-5-8, Hatanodai, Shinagawa-ku, Tokyo 142-8666, Japan; 2 Clinical Research Laboratory, Showa University, School of Medicine, 1-5-8, Hatanodai, Shinagawa-ku, Tokyo 142-8666, Japan

**Keywords:** gastric carcinoma, gastric and intestinal marker, gastric cardia, APC, K-ras, p53, microsatellite instability

## Abstract

Adenocarcinoma of the gastric cardia (C-Ca) is possibly a specific subtype of gastric carcinoma. The purpose of this study was to clarify the differences in the clinicopathological characteristics between C-Ca and adenocarcinoma of the distal stomach (D-Ca), and also the differences in the expressions of gastric and intestinal phenotypic markers and genetic alterations between the two. The clinicopathological findings in 72 cases with C-Ca were examined and compared with those in 170 cases with D-Ca. The phenotypic marker expressions examined were those of human gastric mucin (HGM), MUC6, MUC2 and CD10. Furthermore, the presence of mutations in the APC, K-ras and p53 genes and the microsatellite instability status of the tumour were also determined. C-Ca was associated with a significantly higher incidence of differentiated-type tumours and lymphatic vessel invasion (LVI) as compared with D-Ca (72.2 vs 48.2%, *P*=0.0006 and 72.2 *vs* 55.3%, *P*=0.0232, respectively). Oesophageal invasion by the tumour beyond the oesophago-gastric junction (OGJ) was found in 56.9% of cases with C-Ca; LVI in the area of oesophageal invasion was demonstrated in 61% of these cases. Also, LVI was found more frequently in cases of C-Ca with oesophageal invasion than in those without oesophageal invasion (82.9 vs 58.1%, *P*=0.0197). The incidence of undifferentiated-type tumours was significantly higher in cases with advanced-stage C-Ca than in those with early-stage C-Ca (5 vs 36.5%, *P*=0.0076). A significantly greater frequency of HGM expression in early-stage C-Ca and significantly lower frequency of MUC2 expression in advanced-stage C-Ca was observed as compared with the corresponding values in cases of D-Ca (78.9 vs 52.2%, *P*=0.0402 and 51.5 *vs* 84.6%, *P*=0.0247, respectively). Mutation of the APC gene was found in only one of all cases of C-Ca, and the frequency of mutation of the APC gene was significantly lower in cases of C-Ca than in those of D-Ca (2.4 *vs* 20.0%, *P*=0.0108). The observations in this study suggest that C-Ca is a more aggressive tumour than D-Ca. The differences in biological behavior between C-Ca and D-Ca may result from the different histological findings in the wall of the OGJ and the different genetic pathways involved in the carcinogenesis.

Gastric carcinomas can be subdivided into distal gastric carcinomas and proximal cardiac carcinomas. Although gastric adenocarcinoma remains the major cause of cancer death worldwide, its incidence and mortality appear to have decreased in recent decades (Parkin *et al*, 1988; Boring *et al*, 1991). A striking feature of this decline is the rapid and comparable increase in the incidence of adenocarcinoma of the oesophago-gastric junction (OGJ), for example, adenocarcinomas of the gastric cardia (C-Ca) and of the distal oesophagus (O-Ca)(Blot *et al*, 1991; Pera *et al*, 1993).

It appears that C-Ca may be a specific subtype of gastric carcinoma. It is associated with reflux symptoms, predominance in white males and a greater frequency of differentiated-type tumours as compared with adenocarcinoma of the distal stomach (D-Ca). It has also been described to show a greater tendency towards deeper wall penetration, lymph node metastasis, and a poor prognosis (Kalish *et al*, 1984; Wang *et al*, 1986; MacDonald and MacDonald, 1987; Hamilton *et al*, 1988; Clark *et al*, 1994; Ohno *et al*, 1995; Siewert and Stein, 1996; Kajiyama *et al*, 1997; Pinheiro *et al*, 1999; Tajima *et al*, 2001a). We have previously reported that early-stage of C-Ca is associated with higher frequency of gastric phenotypic marker expression and lower frequency of intestinal metaplasia of the surrounding mucosa as compared with early-stage of D-Ca (Tajima *et al*, 2001a). Recent investigations have also shown that genetic aberrations in the C-Ca gene are probably more closely associated with O-Ca than with D-Ca (El-Rifai *et al*, 2001; Stocks *et al*, 2001). These differences in characteristics between C-Ca and D-Ca suggest that C-Ca might represent a distinct subtype of gastric carcinoma. However, the differences in clinicopathological characteristics, gastric and intestinal phenotypic marker expressions, and genetic alterations between C-Ca and D-Ca have not yet been well clarified.

In this study, we examined the clinicopathological characteristics, gastric and intestinal phenotypic marker expressions and genetic alterations, such as mutations of the APC, K-ras and p53 genes and the microsatellite instability (MSI) status in 72 cases of C-Ca in comparison with those in 170 cases of D-Ca. The purpose of this study was to clarify the differences in the clinicopathological characteristics between C-Ca and D-Ca in the Japanese population, and also the differences with regard to gastric and intestinal phenotypic marker expressions and genetic alterations between the two.

## METHODS

### Patients

Our patient series consisted of 72 patients who had undergone oesophago-gastrectomy for C-Ca between 1998 and 2005 at Showa University Hospital. We also examined 170 patients who had undergone gastrectomy for D-Ca between 2001 and 2005 at the same hospital. For the analysis of the genetic alterations in the tumours, written informed consent was obtained from all of the patients before their participation in this study.

### Histological review

In this study, the macroscopic OGJ was identified as the junction between the end of the tubular oesophagus and the proximal heads of the gastric folds, and tumours with the tumour centre within 2 cm of the macroscopic OGJ were defined as C-Ca (Japanese Gastric Cancer Association, 1998). Resected specimens were fixed in 10% buffered formalin, and the entire length of each lesion was cut into 5-mm-wide sections. All the specimens were embedded in paraffin and processed to obtain 4-*μ*m-thick sections, which were then stained with hematoxylin and eosin (H&E). The histological OGJ and the histological distribution of the tumour were reconstructed on the macroscopic copy of the resected specimens so that the origin of the tumour could be confirmed as being at the gastric cardia and the length of the tumour extension into the oesophagus could be identified. The histological OGJ was identified on the basis of the following histopathological landmarks: the most distal site of detection of oesophageal glands in the submucosal layer, the most distal site of detection of squamous epithelium, and the detection of a double muscularis mucosa in the longitudinally oriented sections (Takubo *et al*, 1991; Tajima *et al*, 1999). When the intramucosal lesion and more than half of the tumour were located in the gastric cardia, the tumour was regarded as a C-Ca.

### Analysis of expressions of phenotypic markers

The following mouse monoclonal antibodies were used: 45M1 (Novocastra Laboratories Ltd, UK), diluted 1:50, to detect HGM; CLH5 (Novocastra Laboratories Ltd), diluted 1:50, to detect MUC6 glycoprotein; Ccp58 (Novocastra Laboratories Ltd), diluted 1:100, to detect MUC2 glycoprotein; 56C6 (Novocastra Laboratories Ltd), diluted 1:40, to detect CD10 glycoprotein expression. 45M1 and CLH5 were examined as the gastric-phenotype markers, and Ccp58 and 56C6 were examined as the intestinal-phenotype markers. HGM is synonymous with MUC5AC and the antibody is known to react with surface foveolar cells in the stomach (Bara *et al*, 1998; Nollet *et al*, 2002). MUC6 glycoprotein is expressed in the mucous cells of the neck zone of the oxyntic mucosa and in the antral glands (De Bolos *et al*, 1995; Reis *et al*, 1999). MUC2 glycoprotein is an intestinal apomucin, which is also known to be expressed in the supranuclear region of the goblet cells in regions of the stomach showing intestinal metaplasia (Kim and Gum, 1995; Reis *et al*, 1999). CD10 glycoprotein is known to be expressed on the brush border of the intestinal epithelial cells (Ronco *et al*, 1984; Trejdosiewicz *et al*, 1985). The avidin–biotinyl–peroxidase complex immunohistochemical method was used for all the immunohistochemical studies, in accordance with a previously described protocol (Hsu *et al*, 1981).

**Figure 1:**
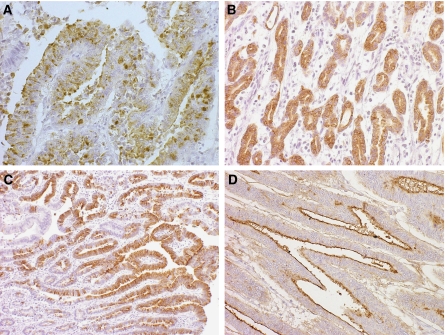
Immunohistochemical analysis of the expressions of phenotypic markers in gastric carcinoma. (**A**) Human gastric mucin (HGM) expressed in the cancer cell cytoplasm (45M1, original magnification × 200). (**B**) MUC6 glycoprotein expressed in the cancer cell cytoplasm (CLH5, original magnification × 100). (**C**) MUC2 glycoprotein expressed in the cancer cell cytoplasm (Ccp58, original magnification × 100). (**D**) CD10 glycoprotein expressed on the luminal surfaces of cancerous glands (56C6, original magnification × 200).

With regard to the evaluations of HGM, MUC6, MUC2 and CD10 staining, distinct staining in more than 5% of the tumour cells was recorded as positive immunoreactivity for the relevant marker (Figure 1). The tumours were classified into four different phenotypes on the basis of the results of the immunohistochemical analysis: tumours with gastric phenotypic cells accounting for more than 5% of the cell population were classified as gastric- (G-) phenotype tumours; those with intestinal phenotypic cells accounting for more than 5% of the cell population were classified as intestinal- (I-) phenotype tumours; those with both gastric and intestinal phenotypic cells accounting for more than 5% of the cell population were classified as gastric and intestinal mixed- (GI-) phenotype tumours; and those with both gastric and intestinal phenotypic cells accounting for less than 5% of the cell population were classified as carcinomas of unclassified- (UC-) phenotype (Tajima *et al*, 2004).

### DNA extraction

Microdissection of 10 *μ*m-thick formalin-fixed, paraffin-embedded serial sections was performed on H&E stained sections for both tumour tissue and normal mucosa. Tissues were precisely microdissected under microscopic visualization using a PixCell laser capture microdissection system (Arcus Engineering, Mountain View, CA, USA) to avoid DNA contamination with that of inflammatory or stromal cell nuclei. Genomic DNA was extracted from the microdissected tissue as described previously (Konishi *et al*, 2004).

### Gene mutation analysis

Mutations of the APC gene in exon 15, codons 1260–1596 and the K-ras gene in codons 12–13 were detected by fluorescence-based PCR-single-strand conformation polymorphism (PCR-SSCP) analysis using primers described previously (Hiyama *et al*, 2002; Lee *et al*, 2002; Yamazaki *et al*, 2006). After amplification, the PCR products from each sample were examined for mutations of the APC or K-ras gene, by fluorescence-based single-strand conformation polymorphism analysis using the ALFexpress DNA sequencer (Amersham Biosciences, NJ, USA) with a cooling bath. Peak patterns were analysed using the ALFwin Fragment Analyzer Programme (Amersham Biosciences), and shifted peaks were defined as mutations in the respective DNA fragment. The nucleotide sequences of the DNA fragments with shifted peaks were determined as described previously. All mutations were reconfirmed by independent PCR reactions and sequencing.

**Figure 2:**
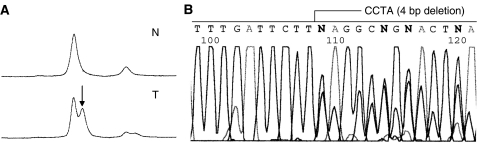
Mutation analysis of the APC gene in gastric carcinoma. (**A**) PCR-single-strand conformation polymorphism (PCR-SSCP) analysis shows a shifted peak (arrowhead) as compared with that for the control normal DNA (T: tumour DNA, N: normal DNA). (**B**) DNA sequence analysis reveals frameshift mutation (CCTA, 4 bp deletion) at codon 1542 of the APC gene.

Mutations of the p53 gene in the exon 5–8 region were detected by fluorescence-based PCR-SSCP analysis using capillary electrophoresis (Makino *et al*, 2000; Yamazaki *et al*, 2006). The nucleotide sequences of the primers have been described previously. Mutations were detected by electrophoresis using a 3100 Genetic Analyzer (Applied Biosystems, CA, USA). The peak pattern was analysed using the QUISCA software (Konishi *et al*, 2004; Yamazaki *et al*, 2006). The nucleotide sequences of the DNA fragments with shifted peaks were determined using the Genetic Analyzer 310 with a BigDye Terminator (Applied Biosystems). All mutations were reconfirmed by independent PCR and sequencing (Figure 2).

### MSI analysis

**Figure 3:**
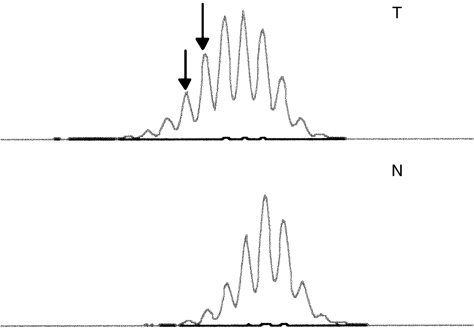
Microsatellite instability analysis in gastric carcinoma. MSI analysis using the BAT-25 marker shows a different allelic shift peak (arrowhead) as compared with that of the control normal DNA (T: tumour DNA, N: normal DNA).

MSI was detected using five microsatellite markers: BAT-25, BAT-26, D2S123, D5S346, and D17S250. The primers and PCR conditions have been described elsewhere (Konishi *et al*, 2004; Yamazaki *et al*, 2006). The samples were subjected to capillary electrophoresis on an ABI 3100 Genetic Analyzer using the Genescan Analysis software (Applied Biosystems). An allelic shift MSI in a microsatellite marker was identified by the presence of at least one additional band in the tumour DNA that was not present in the control DNA. A specimen was considered to be MSI-positive when allelic shift was observed for at least one marker. A tumour sample was considered to contain high-frequency MSI (MSI-H) if two or more of the five informative markers exhibited instability, and to contain low-frequency MSI (MSI-L) when only one marker was unstable. All PCR were repeated on the same sample and only consistent changes in duplicate reactions were scored as abnormalities (Figure 3).

### Statistical analysis

The data were analysed by Student's *t*-test or Mann–Whitney's *U*-test and the *χ*^2^ test or Fisher's exact test. The level of significance was set at *P*<0.05.

## RESULTS

### Clinicopathological characteristics of adenocarcinoma of the gastric cardia and distal stomach

Comparisons of the clinicopathological characteristics between C-Ca and D-Ca are shown in Table 1. C-Ca was associated with more advanced age of the patients and a higher frequency of elevated-type tumours, differentiated-type tumours and lymphatic vessel invasion (LVI) as compared with D-Ca (*P*=0.0406, 0.0067, 0.0010, and 0.0336, respectively).

### Oesophageal invasion and lymphatic vessel invasion in the area of oesophageal invasion in adenocarcinoma of the gastric cardia

**Figure 4:**
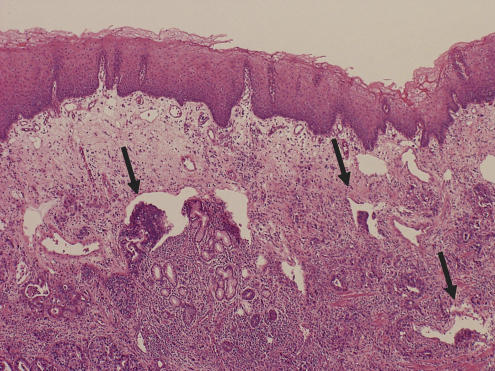
Oesophageal invasion and lymphatic vessel invasion in the area of oesophageal invasion in adenocarcinoma of the gastric cardia. Cancerous cells invading the oesophageal wall under the squamous epithelium and lymphatic vessel invasion are apparent (arrowhead).

Oesophageal invasion by the tumour beyond the histological OGJ was found in 41 of the 72 (56.9%) cases with C-Ca, including five of the 20 (25.0%) with early-stage tumours (tumour invasion limited to the submucosa) and 36 of the 52 (69.2%) with advanced-stage tumours (tumour invasion extending deeper than the muscularis propria). The mean length of oesophageal invasion was 12.8 mm (3.6 mm in the early-stage tumours and 14.1 in the advanced-stage tumours). Among the 41 cases of C-Ca showing oesophageal invasion, LVI in the area of oesophageal invasion was found in 25 (61.0%), including one of the five (20.0%) with early-stage tumours and 24 of the 36 (66.7%) with advanced-stage tumours. In the majority of cases, the LVI in the area of oesophageal invasion was demonstrated in the lamina muscularis mucosa and/or submucosa beneath the non-neoplastic suqamous epithelium of the oesophagus (Table 2A) (Figure 4).

Table 2B shows the incidence of LVI in the cases of C-Ca according to the presence of oesophageal invasion. LVI was found in 18 of the 31 (58.1%) cases of C-Ca without oesophageal invasion (five of the 15 (33.3%) with early-stage tumours and 12 of the 16 (75.0%) with advanced-stage tumours) and 34 of 41 (82.9%) cases of C-Ca with oesophageal invasion [two of five (40.0%) with early-stage tumours and 32 of the 36 (88.9%) with advanced-stage tumours]. The incidence of LVI was significantly higher in the C-Ca cases showing oesophageal invasion than in those without oesophageal invasion (*P*=0.0196).

### Histologic-type of adenocarcinoma of the gastric cardia and distal stomach according to the tumour stage

The histologic type of C-Ca and D-Ca according to the tumour stage is shown in Table 3. Among the 72 cases of C-Ca, differentiated and undifferentiated-type tumours were found in 19 (95.0%) and one (5.0%) of 20 cases with early-stage tumours, respectively, and in 33 (63.5%) and 19 (36.5%) of 52 cases with advanced-stage tumours, respectively. Among the 170 cases of D-Ca, differentiated and undifferentiated-type tumours were found in 40 (58.0%) and 29 (42.0%) of the 69 cases with early-stage tumours, respectively, and 42 (41.6%) and 59 (58.4%) of the 101 cases with advanced-stage tumours, respectively. The prevalence of differentiated-type tumours in the cases of C-Ca was significantly higher than that in the case of D-Ca, among both early- and advanced-stage tumours (*P*=0.0011 and *P*=0.0227, respectively).

Among the 72 cases of C-Ca, the prevalence of undifferentiated-type tumours was significantly higher in the cases with advanced-stage tumours than in those with early-stage tumours (*P*=0.0076). On the other hand, among the cases of D-Ca, although there was a tendency towards a higher prevalence of undifferentiated-type tumours in cases with advanced-stage tumours as compared with that in cases with early-stage tumours, the difference was not significant (*P*=0.103).

### Phenotypic marker expressions in differentiated-type adenocarcinoma of the gastric cardia and distal stomach

Comparisons of the phenotypic marker expressions in differentiated-type C-Ca and D-Ca are shown in Table 4. Expressions of HGM, MUC6, MUC2 and CD10 were demonstrated in 33 (63.5%), 35 (67.3%), 31 (59.6%) and 19 (36.5%) cases of the 52 cases of C-Ca with differentiated-type tumours, respectively, and 46 (56.1%), 48 (58.5%), 64 (78.0%) and 23 (28.0%) cases of the 82 cases of D-Ca with differentiated-type tumours, respectively. The incidence of HGM expression was significantly higher in cases with early-stage C-Ca than in those with early-stage D-Ca (78.9 *vs* 52.2%, *P*=0.0402). The incidence of MUC2 expression was significantly lower in cases with advanced-stage C-Ca than in those with advanced-stage D-Ca (51.5 *vs* 84.6%, *P*=0.0493), and also in all cases with differentiated-type C-Ca than in those with differentiated-type D-Ca (59.6 *vs* 78.0%, *P*=0.0363).

### Genetic alterations in differentiated-type adenocarcinoma of the gastric cardia and distal stomach

Comparisons of the genetic alterations in differentiated-type C-Ca and D-Ca are shown in Table 5. Mutations of the APC, K-ras and p53 genes were detected in one (2.4%), three (7.3%) and 15 (36.6%) of the 52 cases of C-Ca with differentiated-type tumours, respectively, and 16 (20.0%), five (6.3%) and 20 (25.0%) of the 82 cases of D-Ca with differentiated-type tumours, respectively. The frequency of the APC gene mutation was significantly lower in cases with early-stage C-Ca than in those with early-stage D-Ca (0 *vs* 20.3%, *P*=0.0341) and also in all cases with differentiated-type C-Ca than in those with differentiated-type D-Ca (2.4 *vs* 20.0%, *P*=0.0108). There was no significant difference in the frequency of mutations of the K-ras and p53 genes among the patient groups. MSI-L tumours and MSI-H tumours were found in four (9.4%) and one (2.4%) of the 52 cases of C-Ca with differentiated-type tumours, and eight (10.0%) and five (6.3%) of the 82 cases of D-Ca with differentiated-type tumours. There was no significant difference in the MSI status between cases with differentiated-type C-Ca and differentiated-type D-Ca tumours.

## DISCUSSION

C-Ca and D-Ca are characterized by important differences in both aetiological and clinical backgrounds. Several previous reports have described a greater tendency of C-Ca than D-Ca towards deeper wall penetration, lymph node metastasis and poor prognosis, indicating that C-Ca may be a more aggressive tumour than D-Ca (Kalish *et al*, 1984; Wang *et al*, 1986; MacDonald and MacDonald, 1987; Hamilton *et al*, 1988; Clark *et al*, 1994; Ohno *et al*, 1995; Siewert and Stein, 1996; Kajiyama *et al*, 1997; Pinheiro *et al*, 1999; Tajima *et al*, 2001a). In this study, C-Ca was associated with a significantly higher prevalence of LVI than D-Ca. LVI has been regarded as an indicator of tumour aggressiveness in several cancers. The prognostic value of LVI has been demonstrated for diverse tumour entities like gastric carcinoma, adenocarcinoma of the OGJ and squamous cell carcinoma of the oesophagus (Gabbert *et al*, 1992; Brucher *et al*, 2001; von Rahden *et al*, 2005). Therefore, our findings in this study may show the greater aggressiveness of C-Ca than that of D-Ca. Furthermore, in this study, we found specific characteristics of C-Ca in terms of the histological features, phenotypic marker expressions and genetic alterations that might be related to the differences in the biological behavior between C-Ca and D-Ca.

Adenocarcinoma of the OGJ frequently extends across the OGJ to involve both the oesophagus and the stomach. In this study, oesophageal invasion by the tumour beyond the OGJ was frequently (56.9% of the cases) found in cases of C-Ca. In addition, LVI in the lamina muscularis mucosa or submucosa in the area of oesophageal invasion was demonstrated in 61% of C-Ca cases with oesophageal invasion. LVI was more frequently found in C-Ca cases showing oesophageal invasion than in those without oesophageal invasion. Histologically, the oesophageal wall shows significant differences in the structure, especially of the lymphatic and vascular vessels, from the gastric wall. The oesophageal mucosal layer shows an abundance of lymphatic and vascular vessels within a well-developed loose connective tissue layer; the stomach mucosa lacks this characteristic (Goseki *et al*, 1992). Therefore, these specific histological findings in the wall of the OGJ would seem to be important for tumour development in cases of C-Ca through the oesophageal wall and lymphatic vessels. C-Ca may easily invade the oesophagus and lymphatic vessels in the mucosa and submucosa of the oesophagus. The high incidence of the oesophageal invasion and LVI in the area of oesophageal invasion in cases of C-Ca might be one of the reasons for the difference in the incidence of LVI between cases of C-Ca and D-Ca.

C-Ca has been reported to be associated with a higher incidence of differentiated-type tumours as compared with D-Ca (Siewert and Stein, 1996; Tajima *et al*, 2001a). This study revealed that 95% of cases with early-stage C-Ca had differentiated-type tumours. The incidence of differentiated-type tumours was significantly higher in the C-Ca cases than in the D-Ca cases, irrespective of the tumour stage. However, the incidence of undifferentiated-type tumours in cases with advanced-stage C-Ca was significantly higher than that in cases with early-stage C-Ca. On the other hand, such findings were not noted in D-Ca. Therefore, it would seem that in the majority of cases, C-Ca arise as a differentiated-type tumour in the incipient phase of carcinogenesis. In addition, our findings in this study suggest that C-Ca is more likely to transform from differentiated- into undifferentiated-type carcinomas with tumour progression.

Phenotypic marker expressions and genetic alterations during carcinogenesis have been reported to differ markedly according to the histologic-type of tumours (Tajima *et al*, 2001b). In this study, we compared the phenotypic marker expressions and genetic alterations between differentiated-type C-Ca and differentiated-type D-Ca, because the majority of our C-Ca cases, especially those with early-stage tumours, had differentiated-type tumours. Phenotypic marker expressions had initially been investigated to examine the tissue of origin in several cancers, including gastric carcinoma. Among gastric carcinomas, G-phenotype tumours have been reported to account for 27.7% of all differentiated-type tumours, often referred to as intestinal-type tumours by Lauren, whereas I-phenotype tumours have been reported to account for 10.1% of all undifferentiated-type tumours (Tatematsu *et al*, 1990; Egashira *et al*, 1999; Tajima *et al*, 2001a, 2001b, 2004; Yamazaki *et al*, 2006). These previous data suggest that gastric carcinomas may arise from various types of gastric mucosa, although differentiated-type tumours have generally been considered to arise from gastric mucosa with intestinal metaplasia and undifferentiated-type tumours to arise from ordinary gastric mucosa without intestinal metaplasia (Lauren 1965; Nakamura *et al*, 1968). We previously reported that early-stage C-Ca was more significantly associated with G-phenotype tumours, such as tumours showing 45M1 expression and/or class III mucin as detected by paradoxical concanavalin A staining, and more negatively associated with the presence of intestinal metaplasia in the surrounding non-neoplastic mucosa as compared with early-stage D-Ca (Tajima *et al*, 2001a). In this study, the incidence of HGM expression was significantly higher in C-Ca than in D-Ca among early-stage tumours, whereas the incidence of MUC2 expression was significantly lower in cases of C-Ca than in those of D-Ca among the advanced-stage tumours. Therefore, differentiated-type C-Ca may be more strongly associated with the G-phenotype than differentiated-type D-Ca. The phenotypic marker expression pattern has also been reported to be associated with tumour aggressiveness in gastric carcinomas. Even differentiated-type carcinomas of the G-phenotype are more likely to transform into the undifferentiated-type carcinoma and show infiltrative growth to deeper layers of the mucosa or invasion of the surrounding structures through loss of the E-cadherin gene function than tumours of the I-phenotype (Tajima *et al*, 2001b, 2004; Yamazaki *et al*, 2006). Tumours exhibiting such histological transformation have been reported to show more tumour aggressiveness in terms of the invasiveness and propensity for metastasis than other histologic-type tumours (Kozuki *et al*, 2002). Furthermore, G–phenotype tumours have been reported to be significantly associated with a high risk of peritoneal recurrence and a poorer outcome after surgery as compared with tumours of other phenotypes among patients with advanced gastric carcinoma (Tajima *et al*, 2001b, 2004). Therefore, these previous data support our findings in this study that C-Ca predominantly showing the G-phenotype may have a greater tendency towards histological transformation and greater tumour aggressiveness than D-Ca.

Choi *et al* (2000) have reported previously that mutations of the APC/beta-catenin pathway, unlike in colorectal carcinoma, can be identified in only a small subset of patients with OGJ adenocarcinoma. However, to the best of our knowledge, no previous reports describing the association between phenotypic marker expressions and genetic alterations, such as mutations of the APC, K-ras and p53 genes and the MSI status between C-Ca and D-Ca have been published so far. In this study, we confirmed that the APC gene mutation was an extremely rare event in C-Ca, and that the incidence of the APC gene mutation was significantly lower in cases of C-Ca than in those of D-Ca. It has been demonstrated that different genetic pathways according to the phenotypic marker expression pattern of the tumour exist during gastric carcinogenesis (Morohara *et al*, 2006; Yamazaki *et al*, 2006). We have previously reported that chromosomal changes, detected using a comparative genomic hybridisation technique, differ considerably according to the phenotypic marker expression pattern in gastric differentiated-type carcinomas (Morohara *et al*, 2006). We have also demonstrated that the APC gene mutation is a relatively common and early event in I-phenotype tumours, but rather rare in G-phenotype tumours, especially tumours showing HGM expression (Yamazaki *et al*, 2006). Therefore, C-Ca, predominantly showing the G-phenotype, may be associated with a lower frequency of the APC gene mutation. Furthermore, all the findings of the previous and the present study taken together suggest different genetic pathways of carcinogenesis between C-Ca and D-Ca, resulting in the differences in the phenotypic marker expression pattern and biological behavior between the two tumours. Recently, opposing risks of C-Ca and D-Ca associated with *Helicobacter pylori* infection have been reported (Machida-Montani *et al*, 2004; Kamangar *et al*, 2006). Therefore, the authors think that the role of *H. pylori* infection in the carcinogensis may be one of the reasons for the different genetic pathways between C-Ca and D-Ca.

In conclusion, this study shows that C-Ca is significantly associated with a high prevalence of LVI, oesophageal invasion and histological transformation from differentiated- to the undifferentiated-type with tumour progression, indicating that it might be a more aggressive tumour than D-Ca. The differences in the biological behavior between C-Ca and D-Ca may be related to both the specific histological characteristics of the wall of the OGJ and the different genetic pathways of carcinogenesis between the two tumours.

## Figures and Tables

**Table 1:** Clinicopathological characteristics of adenocarcinoma of the gastric cardia and distal stomach

**Variables**	**C-Ca (*n*=72)**	**D-Ca (*n*=170)**	***P*-value**
Age (years)	68.4±10.1	65.0±10.1	0.0406
*Gender*			
Male	55 (76.4%)	111 (65.3%)	NS
Female	17 (23.6%)	59 (34.7%)	
Unknown	0	0	
			
*Macroscopic type*			
Elevated	13 (18.1%)	10 (6.3%)	0.0067
Ulcerative	43 (59.7%)	96 (60.0%)	
Flat	16 (22.2%)	54 (33.8%)	
Unknown	0	10	
Tumour size (mm)	50.1±26.2	57.0±39.8	NS
			
*Histologic-type*			
Differentiated	52 (72.2%)	82 (48.2%)	0.0010
Undifferentiated	20 (27.8%)	88 (51.8%)	
Unknown	0	0	
			
*Depth of invasion*			
M,SM	20 (27.8%)	69 (40.6%)	NS
MP,SS,SE,SI	52 (72.2%)	101 (59.4%)	
Unknown	0	0	
			
*Lymphatic vessel invasion*			
Negative	20 (27.8%)	72 (43.4%)	0.0336
Positive	52 (72.2%)	94 (56.6%)	
Unknown	0	4	
			
*Blood vessel invasion*			
Negative	38 (52.8%)	108 (65.1%)	NS
Positive	34 (47.2%)	58 (34.9%)	
Unknown	0	4	
			
*Lymph node metastasis*			
Negative	30 (41.7%)	93 (55.0%)	NS
Positive	42 (58.3%)	76 (45.0%)	
Unknown	0	1	

C-Ca, adenocarcinoma of the gastric cardia; D-Ca, adenocarcinoma of the distal stomach; M, mucosa; SM, submucosa; MP, muscularis propria; SS, subserosa; SE, serosa exposed; SI, serosa infiltrating adjacent tissue; NS., not significant.

**Table 2A:** Oesophageal invasion and lymphatic vessel invasion in the area of oesophageal invasion in adenocarcinoma of the gastric cardia

**Tumour stage**	***n***	**Number of cases with oesophageal invasion invasion**	**Mean length of oesophageal invasion (mm)**	**Number of cases with lymphatic invasion in the area of oesophageal**
Early	20	5 (25.0%)	3.6 (2–5)	1 (20.0%)
Advanced	52	36 (69.2%)	14.1 (4–75)	24 (66.7%)
Total	72	41 (56.9%)	12.8 (2–75)	25 (61.0%)

**Table 2B:** The incidence of lymphatic vessel invasion in adenocarcinoma of the gastric cardia according to the presence of the oesophageal invasion

	**Lymphatic vessel invasion**			
**Tumour stage**	**C-Ca without oesophageal invasion**		**C-Ca with oesophageal invasion**	
Early	*n*=15	5 (33.3%)	*n*=5	2 (40.0%)
Advanced	*n*=16	12 (75.0%)	*n*=36	32 (88.9%)
Total^a^	*n*=31	17 (58.1%)	*n*=41	34 (82.9%)

C-Ca, adenocarcinoma of the gastric cardia; ^a^*P=*0.0196.

**Table 3:** Histologic type of adenocarcinoma of the gastric cardia and distal stomach according to the tumour stage

**Tumour stage**	**C-Ca (*n*=72)**	**D-Ca (*n*=170)**	***P*-value (AGC *vs* ADS)**
*Early*			
Differentiated	19 (95.0%)	39 (58.0%)	0.0011
Undifferentiated	1 (5.0%)	30 (42.0%)	
			
*Advanced*			
Differentiated	33 (63.5%)	43 (41.6%)	0.0227
Undifferentiated	19 (36.5%)	58 (58.4%)	
			
*P*-value (early *vs* advanced)	0.0076	NS	

C-Ca, adenocarcinoma of the gastric cardia; D-Ca, adenocarcinoma of the distal stomach.

**Table 4:** Phenotypic marker expressions in differentiated-type adenocarcinoma of the gastric cardia and distal stomach

	**C-Ca**			**D-Ca**		
**Phenotypic markers**	**Early (*n*=19)**	**Advanced (*n*=33)**	**Total (*n*=52)**	**Early (*n*=69)**	**Advanced (*n*=13)**	**Total (*n*=82)**
*Human gastric mucin expression* a						
Negative	4 (21.1%)	15 (45.5%)	19 (36.5%)	33 (47.8%)	7 (53.8%)	40 (48.8%)
Positive	15 (78.9%)	18 (54.5%)	33 (63.5%)	36 (52.2%)	6 (46.2%)	42 (51.2%)
						
*MUC6 expression*						
Negative	6 (31.6%)	11 (33.3%)	17 (32.7%)	26 (37.7%)	8 (61.5%)	34 (41.5%)
Positive	13 (68.4%)	22 (66.7%)	35 (67.3%)	43 (62.3%)	5 (38.5%)	48 (58.5%)
						
*MUC2 expression* b						
Negative	5 (26.3%)	16 (48.5%)	21 (40.4%)	16 (23.2%)	2 (15.4%)	18 (22.0%)
Positive	14 (73.7%)	17 (51.5%)	31 (59.6%)	53 (76.8%)	11 (84.6%)	64 (78.0%)
						
*CD10 expression*						
Negative	16 (84.2%)	17 (51.5%)	33 (63.5%)	49 (71.0%)	10 (76.9%)	59 (72.0%)
Positive	3 (15.8%)	16 (48.5%)	19 (36.5%)	20 (29.0%)	3 (23.1%)	23 (28.0%)
						
*Phenotypic marker expression pattern*						
G-phenotype	4 (21.1%)	7 (21.2%)	11 (21.2%)	12 (17.3%)	2 (15.4%)	14 (17.1%)
GI-phenotype	14 (73.7%)	20 (60.6%)	34 (65.4%)	41 (59.4%)	6 (46.2%)	47 (57.3%)
I-phenotype	1 (5.3%)	5 (15.2%)	6 (11.5%)	16 (23.2%)	5 (38.5%)	21 (25.6%)
UC-phenotype	0 (0)	1 (3.0%)	1 (1.9%)	0 (0)	0 (0)	0 (0)

C-Ca, adenocarcinoma of the gastric cardia; D-Ca, adenocarcinoma of the distal stomach.

a *P*=0.0402 (early adenocarcinoma of the gastric cardia *vs* early adenocarcinoma of the distal stomach).

b *P*=0.0493 (advanced adenocarcinoma of the gastric cardia *vs* advanced adenocarcinoma of the distal stomach) and *P*=0.0363 (total adenocarcinoma of the gastric cardia *vs* total adenocarcinoma of the distal stomach).

**Table 5:** Genetic alterations in differentiated-type adenocarcinoma of the gastric cardia and distal stomach

	**C-Ca**			**D-Ca**		
**Genetic alterations**	**Early (*n*=19)**	**Advanced (*n*=33)**	**Total (*n*=52)**	**Early (*n*=69)**	**Advanced (*n*=13)**	**Total (*n*=82)**
*APC gene mutation* a						
Negative	19 (100%)	21 (95.5%)	40 (97.6%)	55 (79.7%)	9 (81.8%)	64 (80.0%)
Positive	0 (0)	1 (4.5%)	1 (2.4%)	14 (20.3%)	2 (18.2%)	16 (20.0%)
Not examined	0	11	11	0	2	2
						
*K-ras gene mutation*						
Negative	18 (94.7%)	20 (90.9%)	38 (92.7%)	65 (94.2%)	10 (90.9%)	75 (93.8%)
Positive	1 (5.3%)	2 (9.1%)	3 (7.3%)	4 (5.8%)	1 (9.1%)	5 (6.3%)
Not examined	0	11	11	0	2	2
						
*P53 gene mutation*						
Negative	16 (84.2%)	10 (45.5%)	26 (63.4%)	53 (76.8%)	7 (63.6%)	60 (75.0%)
Positive	3 (15.8%)	12 (54.5%)	15 (36.6%)	16 (23.2%)	4 (36.4%)	20 (25.0%)
Not examined	0	11	11	0	2	2
						
*MSI status*						
MSS	17 (89.5%)	19 (86.4%)	36 (87.8%)	58 (84.1%)	9 (81.8%)	67 (83.8%)
MSI-L	2 (10.5%)	2 (9.1%)	4 (9.8%)	7 (10.1%)	1 (9.1%)	8 (10.0%)
MSI-H	0 (0)	1 (4.5%)	1 (2.4%)	4 (5.8%)	1 (9.1%)	5 (6.3%)
Not examined	0	11	11	0	2	2

C-Ca, adenocarcinoma of the gastric cardia; D-Ca, adenocarcinoma of the distal stomach.

a *P*=0.0341 (early adenocarcinoma of the gastric cardia *vs* early adenocarcinoma of the distal stomach) and *P*=0.0108 (total adenocarcinoma of the gastric cardia *vs* total adenocarcinoma of the distal stomach).
